# Role of Family Decision-Making and Perceived Social Support in the Mental Health of Mothers of Infants in Rural Western China

**DOI:** 10.1155/da/8794211

**Published:** 2025-11-14

**Authors:** Xiannan Xian, Yuju Wu, Lu Liu, Gary L. Darmstadt, Ann M. Weber, Andrew Rule, Cui Luo, Huan Zhou

**Affiliations:** ^1^Department of Health Behavior and Social Medicine, West China School of Public Health and West China Fourth Hospital, Sichuan University, Chengdu, China; ^2^Institute of Clinical Research, Shenzhen Peking University-Hong Kong University of Science and Technology Medical Center, Shenzhen, China; ^3^Department of Pediatrics, Stanford University School of Medicine, Stanford, California, USA; ^4^School of Public Health, University of Nevada, Reno, Nevada, USA; ^5^Stanford Center on China's Economy and Institutions, Stanford University, Stanford, California, USA; ^6^Department of Medical Education, Liuzhou Traditional Chinese Medical Hospital, Liuzhou, China

**Keywords:** causal mediation analysis, child development, family decision-making, maternal mental health, perceived social support, rural China

## Abstract

**Background:**

The mental health of mothers has an important impact on both the growth and development of infants and on the health of mothers themselves. Family decision-making may play an important role in mother's mental health, yet little research has explored the relationship. This paper explores the association and influential pathways between family decision-making and mental health among mothers of infants in rural western China.

**Methods:**

: Mothers with infants aged 0–6 months in four impoverished counties of a predominantly rural province in southwestern China were enrolled in 2021 from the control group of a cluster-randomized trial using a multistage sampling method and followed up 6 months later. Information on family decision-making, perceived social support, and maternal mental health was collected through household interviews. Causal mediation analysis was employed to explore the association between family decision-making and mental health, and a four-item decomposition was used to explore the control direct effect of family decision-making on mental health, the pure mediation effect of perceived social support, and the possible interaction between exposure and mediation.

**Results:**

Baseline data was collected on 444 mother–infant pairs and 331 were followed up 6 months later. After controlling for the set of confounding factors indicated by our causal framework, the results of longitudinal causal mediation analyses showed that family decision-making was negatively associated with depression, anxiety, and stress after controlling for confounding factors (*β*_total effect_: −1.323, −0.928, and −1.351, respectively). Perceived social support played a proportional mediating role in each of the above associations (pure indirect effects of 22.33%, 22.60%, and 27.02%, respectively), while interaction effects were not significant.

**Conclusions:**

Family decision-making had a direct negative effect on maternal depression, anxiety, and stress, and perceived social support played a mediating role. These findings can be used to guide future interventions to improve maternal mental health and healthy child development in rural China.

## 1. Introduction

In recent years, maternal mental health has received increasing public health attention. The World Health Organization (WHO)/UNICEF Nurturing Care Framework highlights improving parental mental health as a priority for promoting healthy social and emotional development in young children, preventing childhood diseases, and reducing parental morbidity, especially in low- and middle-income countries (LMIC) [1]. The psychological state of mothers can directly affect their parenting abilities and behaviors, potentially impeding stimulating interactions between parent and child [2] and increasing the likelihood of developmental delays [3]. Moreover, studies have shown that mental health conditions such as depression and anxiety in mothers can have a negative impact on the ability of infants to regulate their own emotional and behavioral state [4], whereas a healthy, well-regulated parental psychological state can positively contribute to the early development of children [5].

Mental health conditions are common among mothers worldwide. In a 2022 report, the WHO [6] estimated that more than 10% of women experience mental disorders such as depression or anxiety after childbirth. Mental health conditions are especially common among mothers in LMICs, where rates of perinatal depression and anxiety among mothers are two to three times higher than among mothers in high-income countries [7]. In LMICs, about 19% of mothers are estimated to experience depression during pregnancy and the postpartum period [8], and about 24% experience anxiety symptoms [9].

Understanding factors that may influence maternal mental health is important for developing interventions to support better postpartum mental health and nurturing child care. Previous studies have revealed that gender inequality, lack of health insurance, intimate partner violence, young age, and low educational attainment are all important risk factors for low mental health status among mothers in LMICs [10–13], and that perceived gender inequality during pregnancy is associated not only with maternal depression but also with child emotional and behavioral problems [14, 15]. One factor that has received particular attention in the literature is perceived social support, or one's own subjective emotional experience and feeling of satisfaction around being respected, understood, and supported by others [16]. In related research on social support theory, it is found that perceived social support can have an impact on mental health by influencing aspects such as individual emotional regulation, cognitive evaluation, and behavioral coping [17, 18]. Perceived social support has been associated with improved mental health, including lower levels of depression, anxiety, and stress in mothers [19–23] and has been shown to act as a mediator in a variety of mental health contexts [24–28].

According to ecosystem theory, an individual's social environment is an ecosystem in which their interaction with the social environment and their behavior and psychology will be influenced by the social environment [29]. The microsystem is a component of the ecosystem and an immediate environment for an individual's activities and interactions, with the family central to the microsystem. The interaction between an individual and the family will have an impact on an individual's behavior and psychology.

Family decision-making, or the degree to which one is involved in making decisions about one's own life or family affairs [30], has been widely recognized as a core dimension of women's empowerment and autonomy, alongside financial control, freedom of movement, and access to resources [31, 32]. Family decision-making may also play an important role in maternal mental health, but it has received less attention in the literature to date. Previous research on family decision-making has largely focused on the effects of female decision-making within the family on intra-family relationships [33, 34], as well as its effects on infant health and development [35–38]. In addition, a study of older adults in India found that participation in family decision-making improved mental health and subjective well-being [39]. However, few studies to date have investigated the associations between family decision-making and mental health among mothers in LMICs. In China, family structures—such as multigenerational co-residence and gender-based divisions of labor—may shape women's participation in household decision-making [40]. Therefore, examining family decision-making in this sociocultural context is of particular relevance.

Moreover, within the framework of ecosystem theory, perceived social support also originates from supportive relationships within the microsystem, particularly emotional and interactive exchanges among family members. These interactions are central to the individual's immediate environment and contribute to subjective experiences of being understood, respected, and supported—key dimensions of perceived social support [29, 41]. Given that family decision-making primarily occurs at this level, perceived support from close family relationships may serve as an important psychological mechanism linking family dynamics to maternal mental health outcomes. Although previous studies have shown that perceived social support can mediate the associations between individual, family, or social factors and maternal mental health outcomes such as depression and anxiety [27, 42, 43], no research to date has examined whether it specifically mediates the relationship between family decision-making and maternal mental health.

It is particularly urgent to clarify these associations in rural China. First, as one of the world's largest low- and middle-income settings, rural China can provide valuable evidence that can be used to guide future maternal mental health interventions in LMICs. Second, the prevalence of poor mental health among maternal caregivers in rural China is at a critical level. Previous studies conducted in rural China have estimated that maternal depression rates range from 15.6% to 39.9% [44–46], anxiety rates range from 15.3% to 32.9% [47, 48], and stress rates range from 9.0% to 12.7% [49]. Third, despite these high rates of poor mental health and the fact that family decision-making is a possible important influencing factor, few studies to date have investigated the associations between family decision-making and mental health among mothers in rural China, with most research in this area focusing instead on the effects of family decision-making on marital satisfaction, household assets, or improving household livelihoods [33, 34]. While the literature on perceived social support and mental health among women in rural China is comparatively more robust [19–23], the specific effects of family decision-making on maternal mental health and the potential for perceived social support to mediate effects of family decision-making on maternal mental health remain under-researched in rural China.

The primary aim of this study is to clarify the associations between family decision-making, perceived social support, and mental health among mothers with infants in rural China. To do so, we pursue three specific objectives. First, we measure the incidence of depression, anxiety, and stress among mothers to assess their mental health status. Second, we measure the association between involvement in family decision-making and mental health. Finally, we use a causal mediation framework to identify the extent to which perceived social support mediates this association and decompose the total mediation effect to determine how much of the mediation by perceived social support can be explained by pure mediating effects and how much, if any, can be explained by interaction effects between perceived social support and family decision-making.

## 2. Methods

### 2.1. Sample Selection

This study was nested within the Healthy Future program, a stratified, multistage, cluster-randomized controlled trial conducted in a predominantly rural prefecture of Sichuan Province in southwestern China. Of the prefecture's nine counties, seven had been designated as national or provincial poverty-stricken counties. The sample was selected in 2021 using a multi-stage cluster random sampling design. In the first stage, four of the seven poverty-stricken counties were randomly selected. In the second stage, all townships within these counties were included, except for urban townships and those with a population below 10,000, yielding a total of 119 eligible townships. In the third stage, lists of families—either with pregnant women at ≥13 weeks of gestation or infants aged 0–6 months—were obtained from local health centers in the sampled townships. Among the 119 townships, 40 were randomly assigned to receive a parenting intervention, and the remaining 79 served as the control arm without intervention. We analyzed data only from the control arm, as the intervention included content on maternal mental health that could influence psychological outcomes. Although the control group included both pregnant women and postpartum mothers at baseline, our analysis focused exclusively on mothers who had already given birth.

Within the 79 control townships, families with infants aged 0–6 months were identified through local health centers in 2021. Families were excluded if the infant was younger than 3 weeks or had severe health conditions (e.g., malignancies, brain injuries, or serious infectious diseases such as sepsis, tuberculosis, or measles). A total of 462 eligible mother–child dyads were enrolled. The baseline survey included 444 mothers. Due to the impact of the COVID-19 pandemic, some participants had relocated or were unwilling or unable to participate in follow-up face-to-face interviews because of health concerns or family-related circumstances. As a result, the follow-up survey conducted in 2022 successfully completed interviews with 331 mothers.

The data used in this study were drawn directly from the original trial's baseline and follow-up surveys, with no additional data collection or intervention. As the control group received no intervention by design, this secondary analysis did not affect the conduct or outcomes of the parent trial.

A detailed description of the parent trial design is available in a previously published protocol [50].

### 2.2. Date Collection

A structured questionnaire was digitized using the World Bank's Survey Solutions platform. Approximately 60 interviewers, all undergraduate or graduate students from medical universities in Sichuan province, were recruited and received centralized training delivered by project team members. The training covered the study background, objectives, questionnaire content, and interview procedures, and also incorporated role-play exercises simulating interviewer–respondent interactions to standardize data collection. In the control group from which our data were drawn, data collection was conducted by a subset of these interviewers through face-to-face tablet-based interviews in quiet and private settings, with efforts to minimize the presence of other household members. On average, each interview lasted about 90 min. Written informed consent was obtained from all participants. Data quality was ensured through built-in logic checks, daily interviewer cross-checks, and supervisor review with follow-up verification when necessary. Data were securely stored on a cloud-based server with restricted access.

### 2.3. Patient and Public Involvement

Patients and the public were not involved in the design, recruitment, conduct, or dissemination plans of this research.

### 2.4. Measures

#### 2.4.1. Mothers' Depression, Anxiety, and Stress

Maternal depression, anxiety, and stress were used as dependent variables and were measured during the 6-month follow-up survey using the Depression Anxiety Stress Scale-21 (DASS-21). DASS-21, a streamlined version of the DASS developed by Lovibond [51], has been widely used internationally and in China and has been shown to have good reliability and validity in screening for depression, anxiety, and stress [52–54]. The scale is comprised of three subscales corresponding to depression, anxiety, and stress, each of which contains seven specific items. Participants rated how much each symptom had applied to them over the past week, in accordance with the DASS-21 guidelines. Each item is scored on a 4-point scale from 0 to 3, with 0 indicating “very low frequency of symptoms” and 3 indicating “very high frequency of symptoms.” The score for each subscale is obtained by summing the scores of the seven items, then multiplying the sum by 2 to align with the 1995 version of the scale [55]. DASS-21 has a possible range of 0–42 [51]. Following Lovibond, we determined that depression, anxiety, or stress was present when the score on the depression, anxiety, or stress scale was greater than 9, 7, or 14, respectively. Cronbach's alpha for the DASS-21 total scale and depression, anxiety, and stress subscales were 0.93, 0.88, 0.72, and 0.84, respectively.

#### 2.4.2. Perceived Social Support

This study used perceived social support as a mediating variable, which was measured at baseline. Perceived social support was measured using the Multidimensional Scale of Perceived Social Support developed by Zimet, which quantifies the degree of support that individuals perceive that they receive from family, friends, and other sources [56] on a scale from 1 indicating “very unsupportive” to 7 “very supportive.” Possible scores ranged from 12 to 84, with higher overall scores indicating higher levels of social support felt by the individual. Based on established cutoffs, scores of 12–35 indicate low perceived support, 36–60 indicate medium perceived support, and 61–84 indicate high perceived support [57]. Validation studies have shown that both the scale and its Chinese translation have good reliability [58, 59]. Cronbach's alpha for this scale was 0.91.

#### 2.4.3. Family Decision-Making

Family decision-making was used as the independent exposure variable and was measured at baseline. As there is no generally agreed-upon scale for the measurement of family decision-making, we constructed a set of items based on decision-making modules from prior studies conducted in LMICs. Specifically, we referred to the instruments developed by Shroff et al. [31] and Peterman et al. [60], which were originally validated in South Asian and African contexts. These instruments typically include decision-making domains related to household consumption, maternal personal affairs, and child care. To adapt the measurement to the rural Chinese context, we reviewed the decision-making items used in those studies, selected 10 items that were most relevant and culturally appropriate, and ensured balanced coverage across domains. Appropriateness was judged by conceptual alignment, salience to common rural household practices, and clarity and acceptability of wording. Language was first refined by the study team and then reviewed through pilot testing with rural mothers and feedback from interviewers, ensuring contextual relevance. The final questionnaire included 10 questions to assess which member of the household was primarily responsible for making decisions across three categories of family decisions: decisions about family matters (e.g., “what food to buy for meals at home”), decisions about the mother's personal matters (e.g., “whether to work outside the home to earn money”), and decisions about caring for young children (e.g., “what to do if the baby is sick”). In total, the survey contained 10 family decision questions, with each question scored on a scale of 0–2. A subscore of 2 indicated that the decision was usually made by the respondent; a subscore of 1 indicated that it was usually decided jointly with other family members; and a subscore of 0 indicated that it was usually decided entirely by other family members. All subscores were then added together to obtain a total family decision-making score, with a possible range of 0–20. Higher scores indicated that the respondent had more involvement in making household decisions. The specific scale items used to evaluate family decision-making in this study can be found in Supporting Information 1: Table S1. Exploratory and confirmatory factor analyses supported a three-factor structure, and the model demonstrated good fit (e.g., CFI = 0.955, RMSEA = 0.048). The scale showed acceptable internal consistency (Cronbach's α = 0.689). Detailed results are presented in Supporting Information 2: Tables S2–S5.

Our index was designed to estimate the respondent's involvement in family decision-making but not the equality of the decision-making process, so that a higher score does not necessarily imply that the burden of decision-making is equally shared between partners. Decision-making equality, while outside the scope of the current paper, might be explored in future studies using derivations of this scale.

### 2.5. Potential Confounders

To select appropriate covariates, we identified potential confounding variables from previous studies [61–65] and used the evidence synthesis method for constructing directed acyclic graphs (DAGs) using DAGitty v3.1 software. DAG can be used to determine which confounding variables should be controlled for in a study when exploring the influence of one variable on another based on the “backdoor criterion” [66]. In the analysis model of this study, we treated infant sex, number of siblings, mode of birth, mother's age, marital status, occupation, education, work experience outside the home, whether the mother's natal family is in the township, whether the father lives together with the mother, number of adults at home, and family assets as covariates. Among these, number of siblings, number of adults at home, and family assets were also considered as proxy indicators reflecting certain structural features of the household, such as family composition and economic capacity.

Family assets were determined by ascertaining how many of a preset list of fixed assets were owned by the household, including water heater, washing machine, computer, refrigerator, air conditioner, motorcycle, and automobile. The family asset index was calculated based on this list using principal component analysis and used to group family assets into quartiles.

### 2.6. Statistical Analysis

**Figure 1:**
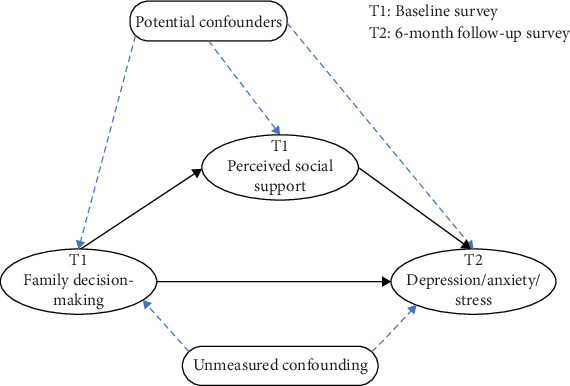
Relationship between variables of the hypothetical model.

Traditional mediation methods cannot identify interaction effects between exposure and mediator and do not make explicit the necessary assumptions for causal mediation estimation. Therefore, this study takes a causal mediation approach to estimate the effect of an exposure (i.e., family decision-making) on maternal mental health in the presence of a potential mediator (i.e., perceived social support) that may interact with family decision-making, as shown in Figure 1. As described by VanderWeele, in addition to requiring consistency, positivity, and no interference, the identification of causal mediating effects also requires the following four assumptions to be satisfied [67]: (i) no unmeasured confounders exist between the exposure and outcome; (ii) no unmeasured confounders exist between mediator and outcome; (iii) no unmeasured confounders exist between exposure and mediator; and (iv) the mediator outcome confounders are not themselves affected by the exposure.

Under this set of assumptions, causal mediation analysis assesses the proportion of the total effect (TE) that is attributable to the exposure alone, the mediated pathway, and the interaction of the two factors. The decomposed effects in this framework are given in Equations (1)–(3) [67]:

(1) $$\begin{array}{c} \text{TE}=\text{PNDE}+\text{TNIE}, \end{array}$$

(2) $$\begin{array}{c} \text{PNDE}=\text{CDE}+\text{RIE}, \end{array}$$

(3) $$\begin{array}{c} \text{TNIE}=\text{MIE}+\text{PIE}. \end{array}$$

In this study, TE is the total effect; PNDE is the pure natural direct effect of the exposure, or the direct effect of family decision-making on maternal mental health; TNIE is the total natural indirect effect, or the indirect effect of family decision-making on maternal mental health; CDE is the controlled direct effect, or the effect of family decision-making on maternal mental health when controlling for perceived social support; RIE is the reference interaction effect, or the combined effect of family decision-making and perceived social support on maternal mental health in the absence of a mediation effect; MIE is the mediator interaction effect, or the combined effect of family decision-making and perceived social support on maternal mental health in the presence of a mediating effect; and PIE is the pure indirect effect, or the effect of family decision-making on maternal mental health from the perceived social support only in the absence of a direct effect of the exposure.

### 2.7. Data Analysis

Continuous variables were described statistically by the mean and standard deviation (SD) or interquartile range (IQR), and categorical variables were described by percentage (%) or proportion. Correlations between variables were examined using Pearson correlation analysis. Causal mediation analysis was performed in Stata using *med4way* commands for longitudinal data [68] to explore the associations between family decision-making and depression, anxiety, and stress, and the mediating role of perceived social support for each mental health measure. Finally, we conducted sensitivity analyses to see if our results were replicated in subsamples of younger (≤11 months) and older (≥12 months) infants at the 6-month follow-up. Causal mediation was conducted under the assumption of no unmeasured confounding. The sensitivity analyses assessed how robust the results about direct and indirect effects were to violations in the unmeasured confounder assumptions being made [69, 70]. If the results were inconsistent, we repeated the causal mediation analyses of the final model, adjusting for the available covariates and infant age (younger vs. older) [71]. Additionally, we computed *E*-values to quantify how strongly the “no unmeasured confounding” assumption would need to be violated to explain away the observed effects. The *E*-value is the minimum association, on the risk-ratio scale, that an unmeasured confounder would need with both the exposure and the outcome, conditional on measured covariates [72]. As an additional robustness check, we re-coded the decision-making index into a binary scale (0 = others decide; 1 = joint or mother alone) and repeated the analyses to assess whether findings were sensitive to alternative codings of the exposure.

## 3. Results

### 3.1. Sociodemographic Characteristics of the Study Population

Among the 444 infants in the baseline survey, 227 (51.1%) were female and 171 (38.5%) were delivered vaginally (Table 1). Among the 444 mothers in the baseline survey, 228 (65.4%) were aged 21–30 years, 437 (98.4%) were married, and 191 (43.0%) had graduated from junior high school. In half of the families (*n* = 224, 50.5%), the father lived together with the mother. In the follow-up survey of 331 mother–infant pairs, except for infant age, there were no statistical differences in demographic characteristics between the baseline and follow-up samples (Table 1).

### 3.2. Family Decision-Making, Perceived Social Support, and Mental Health Scores and Correlations of Each Dimension

The proportions of mothers at risk of depression, anxiety, and stress at the follow-up were 17.8%, 15.7%, and 10.6%, respectively (Table 2). Baseline mean scores for family decision-making was 14.39 (SD = 3.28), indicating a moderately high level of involvement in household decisions (possible range: 0–20). The mean score for perceived social support was 63.15 (SD = 13.08), which falls within the high range of perceived support. While Table 2 presents median and IQR values for the mental health outcomes due to non-normal distributions, we additionally report mean scores to facilitate interpretation. The mean (SD) scores for depression, anxiety, and stress were 3.98 (5.64), 3.44 (4.14), and 6.17 (6.36), respectively. Perceived social support and family decision-making were negatively correlated with depression, anxiety, and stress. Depression, anxiety, and stress subscales were positively correlated with each other.

### 3.3. Causal Mediation Analysis

The *β* values for the TE of family decision-making on depression, anxiety, and stress were −1.323 (95% CI: −1.750 to −0.896), −0.928 (95% CI: −1.405 to −0.451), and −1.351 (95% CI: −1.826 to −0.876), respectively, indicating that those with high family decision-making scores were less likely to experience depression, anxiety, and stress than those with low family decision-making scores (Table 3). This TE was composed of direct and indirect effects that were decomposed into four effect estimates.

Among the direct effects, the controlled direct effects (CDEs) of the three models were *β* = −1.035 (95% CI: −1.437 to −0.633), −0.752 (95% CI: −1.173 to −0.331), and −1.029 (95% CI: −1.429 to −0.629), indicating that, after holding the mediator constant, higher levels of family decision-making were associated with reduced likelihood of depression, anxiety, and stress. All RIE 95% CIs included zero, suggesting that the combined effect of family decision-making and perceived social support on mental health (interaction-induced effects only) was statistically insignificant and negligible, in the absence of a mediating effect through perceived social support.

Among the indirect effects, the MIE 95% CIs for all three models included zero, indicating that the combined effect of family decision-making and perceived social support on mental health (the effect caused by the combination of interaction and mediation) was statistically insignificant and negligible in the presence of mediation through perceived social support. The PIE estimates were −0.295 (95% CI: −0.482 to −0.108), −0.210 (95% CI: −0.354 to −0.065), and −0.365 (95% CI: −0.579 to −0.151), indicating that family decision-making, when mediated through perceived social support, was associated with lower risk of depression, anxiety, and stress, in the absence of a direct effect of decision-making. The percentages of the TE explained by the PIE were 22.3%, 22.6%, and 27.0%, respectively. The percentages of the TE explained by the NIE were 21.3%, 16.2%, and 21.5%, respectively.

Results of sensitivity analyses conducted in different infant age groups are shown in Supporting Information 2: Tables S6–S8. In the younger group, the direction and significance of both the TE and the CDE are supported. However, in the older group, the effects are not significant. To remove potential biases associated with age changes, we iterated the final analysis and adjusted for the available covariates and infant age (0 = younger, 1 = older). The direction and significance of the associations remained the same as in Table 3. As a complementary check for unmeasured confounding, the E-values for the TE were 6.97 (depression), 4.50 (anxiety), and 7.19 (stress), indicating that—conditional on measured covariates—only an unmeasured confounder associated with both the exposure and the outcome by risk ratios of at least these sizes could explain away the observed effects. Full calculations (including the CI-limit *E*-values) are provided in Table S9. As an additional robustness check, when the decision-making index was re-coded into a binary scale, the direction and significance of the associations remained consistent with those in Table 3 (Supporting Information 2: Table S10), suggesting that our findings are robust to alternative codings of the family decision-making index.

## 4. Discussion

Our results build on previous studies about maternal mental health in LMIC settings and offer new insights into the specific mechanisms by which family decision-making and perceived social support impact maternal mental health status. We found that while mothers in rural China face high risks of depression, anxiety, and stress, these risks are significantly higher for mothers who have less involvement in family decision-making. Moreover, perceived social support acts as a mediator in the negative association between family decision-making and mental health conditions, and its impact comes primarily from pure mediating effects rather than interaction effects with family decision-making. This relationship between perceived social support, family decision-making, and mental health has not previously been reported in the literature and has major implications for future interventions to promote maternal mental health and, by extension, healthy child development in rural China. Given the central role of family decision-making in our findings, it is worth noting that in our study, the average family decision-making score was 14.39 out of 20, indicating a moderately high level of maternal involvement in household decisions. This may reflect the specific socio-cultural and programmatic context of the study setting. While this level of decision-making agency may contribute to the observed associations with maternal mental health, it may also limit the generalizability of our findings to populations where women have similar levels of involvement in household decision-making.

Although our results confirm that negative mental health conditions are prevalent among mothers in rural China, they also reveal that family decision-making—a social variable subject to change—plays an important role. The proportions of mothers at risk of depression, anxiety, and stress in our sample (17.8%, 15.7%, and 10.6%, respectively) were higher than global estimates, slightly lower than pooled estimates from LMICs, and closer to the lower end of the ranges previously reported in rural China [44, 49, 73]. While previous research has found family decision-making to be an influential factor in mental health [74, 75], our analysis extends this understanding by identifying both direct and indirect associations between family decision-making and maternal mental health in rural western China. There are both psychological and economic mechanisms that might help explain this association. For instance, as family decision-making is associated with gender equality and a lower likelihood of experiencing domestic violence [76], it is possible that mothers with greater involvement in family decision-making may have improved mental health outcomes because of greater feelings of agency, emotional security, safety, or self-worth. At the same time, it is also possible that increased participation in family decision-making increases mothers' sense of economic security and of being in control of their financial situation, which is correlated with improved mental health outcomes [39]. This interpretation is further supported by the composition of our family decision-making score, which includes several items related to financial decisions—such as decisions about household spending, large purchases, and employment. Further research should explore these and other possible reasons for the association between family decision-making and mental health.

Our evidence of a direct relationship between family decision-making and maternal mental health can be used to design future policy interventions targeted at improving maternal mental health in rural China. For instance, future interventions might focus on empowering mothers to actively participate in making decisions on important family matters, including but going beyond decisions about child care, for example, household decisions about purchases, finances, educational pursuits, and leisure activities, thereby increasing their feelings of financial security and improving their psychological health. Evidence from other resource-constrained settings supports this direction. For example, a community-based intervention in Zimbabwe that engaged women and men in gender-synchronized group discussions not only increased women's participation in family decision-making but also significantly reduced the incidence of maternal depression [77]. At the same time, our results should be interpreted carefully in the context of a study conducted in China by Li [78], which found that the relationship between family decision-making and women's happiness is partly dependent on social norms. Under heavily patriarchal social norms, this previous study found that the women who self-reported having more power over important family and financial decisions in the household did not experience a positive impact on their happiness, and interventions in their family decisions could be ineffective or even have harmful outcomes. Notably, however, in examining the effects of policies (e.g., tuition-free primary education, paid maternity and parental leave) on maternal and child health, using family decision-making as a proxy for gender norms, Heymann et al. found that exposure to these pro-equality policies substantially increased women's and their children's likelihood of having better health outcomes while also increasing women's likelihood of having sole or joint decision-making power [34]. While our findings provide strong evidence that targeting family decision-making is a promising direction for future interventions, it is important to note that this study did not include direct measures of family structure or intra-family dynamics, such as family values, household organization, or communication patterns. As such, these interventions will need to be carefully interpreted and designed in light of both the social norms of the target population and the broader family context, in order to avoid unintended negative effects on maternal mental health. Future research is warranted on the effects of laws and policies on improving family decision-making, and qualitative research into caregiver (maternal and paternal) attitudes toward shared or equitable caring responsibilities might aid in the design of these interventions. It is important to note that our family decision-making score reflects maternal involvement across household decisions, with autonomous choices scored higher than joint ones to approximate deeper participation in decision-making. However, this may not fully capture the nuances of joint decision-making. Future work should refine this measure to better distinguish between autonomous and collaborative forms of decision-making, particularly in culturally diverse settings.

A second major finding of this paper is that perceived social support mediates 22.3%, 22.6%, and 27.0% of the association between family decision-making and maternal depression, anxiety, and stress, respectively. As the (insignificant) interaction effects between family decision-making and perceived social support were decomposed and excluded in the analysis, the impact of perceived social support in this relationship was shown to mostly stem from a pure mediating effect. While previous studies have reported that perceived social support has an important influence on mental health [79, 80], we extend this finding by revealing the mediating role of perceived social support between family decision-making and mental health. This finding should also be incorporated into future interventions to improve maternal mental health in rural China. As perceived social support includes subjective feelings of support from family, friends, and other significant relationships that buffer external pressures and negative subjective feelings [81], it is important to acknowledge that mothers may face limitations in accessing such support through their existing social networks—due to individual characteristics, willingness to seek help, and other interpersonal factors. Therefore, future interventions should also consider strengthening institutional or community-based sources of support to enhance perceived support and reduce the risk of maternal mental health problems. For example, a recent cluster-randomized trial in Ethiopia showed that community-based psychoeducation sessions during pregnancy significantly enhanced mothers' perceived social support, highlighting the potential of institutional approaches to reduce maternal mental health risks [82].

In addition, we conducted a sensitivity analysis to assess the robustness of our main findings to potential unmeasured confounding. Infant age at follow-up was included as a covariate, given that maternal stress, emotional state, and perceived support may vary with infant age due to evolving caregiving demands—such as sleep disruption, the introduction of complementary foods, and changing maternal roles during the first year postpartum [83]. Results remained consistent, supporting the stability of the observed associations. In addition, *E*-value calculations indicated that only unmeasured confounders of substantial magnitude could explain away the observed effects, and analyses with a binary coding of the family decision-making index yielded results consistent in both direction and significance with the three-level specification. Collectively, these sensitivity analyses provide further evidence for the robustness of the main findings.

## 5. Strengths and Limitations

Several strengths of this study set it apart from most previous research on the determinants of maternal mental health in LMIC settings. First, this study was a longitudinal follow-up survey using a longitudinal causal mediation model for analysis. By using two-wave (baseline and follow-up) data, the independent variable and the dependent variable have a temporal sequence. DAGs were used to identify confounding factors and control them in the analysis of the model to enhance causal inference. Second, most existing studies of the determinants of maternal mental health decomposed the TE only into a direct and indirect effect without considering the interaction effect, which makes it difficult to estimate the mediating effect comprehensively and accurately. The present study included interaction effects in the analysis, and although the results show that the interaction effects were not significant, PNIEs can be seen after excluding the interaction.

However, three limitations warrant mention. First, causal mediation analysis rests on the assumption that all confounding factors have been controlled for [84]. In this study, a set of key confounding variables was identified through DAGs. In the model analysis, the effects of these confounding variables were controlled. Although it cannot be guaranteed that all potential confounding factors have been fully considered, the robustness of the results was tested through sensitivity analysis. Secondly, the study did not include direct measures of family functioning or internal structure (e.g., communication styles, role distributions, or cohesion). While proxy indicators such as the number of siblings, adults in the household, and household assets were included, these cannot fully capture internal dynamics. Future research should incorporate validated tools to better evaluate the role of family functioning in maternal decision conflict and mental health. Finally, as this study used self-reported measures, reporting bias may have influenced some results. In addition, the assessment of maternal mental health symptoms referred to the past week and may therefore have been subject to minor recall bias. Future research should continue to seek more standardized and bias-resistant ways to measure decision-making, social support, and mental health. For family decision-making in particular, it may be useful to incorporate reports from multiple household members—such as fathers, grandparents, or other caregivers—to reduce single-informant bias. Observational or interaction-based approaches may also complement self-reports and offer more objective insights into family decision-making dynamics.

## 6. Conclusions

Despite these limitations, the present study presents strong evidence that among mothers of infants in rural China, maternal participation in family decision-making can have a positive direct effect on maternal mental health, as well as a positive indirect effect on maternal mental health via perceived social support. Family decision-making and perceived social support are therefore key factors for researchers and policymakers to focus on in their attempts to address the widespread negative mental health status of mothers in rural China. Future interventions can target family decision-making and perceived social support to encourage mothers to actively participate in decision-making in family matters, actively benefit from the support from family and friends, and enjoy positive mental health outcomes, thus promoting healthy child growth and development.

## Figures and Tables

**Table 1:** Sociodemographic characteristics of infants and mothers.

Characteristic	Baseline (*N* = 444)		6-month follow-up (*N* = 331)		*t/χ* ^2^	*p*
	Number	Percentage (%)	Number	Percentage (%)		
Infant age (months)	2.66 ± 1.72		11.77 ± 2.42		61.23	<0.001
Infant sex					0.034	0.853
Female	227	51.13	164	49.55	—	—
Male	217	48.87	167	50.45	—	—
Infant delivery					0.092	0.764
Vaginal	171	38.51	131	39.58	—	—
Cesarean	273	61.49	200	60.42	—	—
Number of infant's siblings					0.372	0.946
0	157	35.36	111	33.53	—	—
1	238	53.60	181	54.68	—	—
2	49	11.04	39	11.78	—	—
Maternal age (years)					0.374	0.946
≤20	10	2.25	6	1.81	—	—
21–30	288	64.86	212	64.05	—	—
31–40	137	30.86	105	31.72	—	—
≥41	9	2.03	8	2.42	—	—
Marital status					0.064	0.800
Married	437	98.42	325	98.19	—	—
Others (divorce, widowhood, etc.)	7	1.58	6	1.81	—	—
Highest education					0.353	0.950
Primary school and below	78	17.57	61	18.43	—	—
Middle school	191	43.02	145	43.81	—	—
High school	107	24.10	74	22.36	—	—
University and above	68	15.32	51	15.40	—	—
Occupational status					0.347	0.556
Unemployed	381	85.81	279	84.29	—	—
Employed	63	14.19	52	15.71	—	—
Whether the mother's natal family is in this township					0.095	0.758
Yes	211	47.52	161	48.64	—	—
No	233	52.48	170	51.36	—	—
Mothers' work experience outside					0.063	0.803
Yes	389	87.61	288	87.01	—	—
No	55	12.39	43	12.99	—	—
Whether father lives with the mother					0.028	0.867
Yes	224	50.45	169	51.06	—	—
No	220	49.55	162	48.94	—	—
Number of adults at home					0.219	0.994
2	19	4.28	16	4.83	—	—
3	79	17.79	61	18.43	—	—
4	123	27.70	91	27.49	—	—
5	146	32.88	106	32.02	—	—
6	77	17.34	57	17.22	—	—
Family assets					1.017	0.797
Low level	10	2.25	8	2.42	—	—
Lower-middle level	206	46.40	151	45.62	—	—
Upper-middle level	150	33.78	105	31.72	—	—
High level	78	17.57	67	20.24	—	—

**Table 2:** The scores of continuous variables and correlation between variables (*N* = 331).

Variables	Perceived social support mean ± SD	Family decision-making mean ± SD	Depression M (P25, P75)	Anxiety M (P25, P75)	Stress M (P25, P75)
Scores	63.15 ± 13.08	14.39 ± 3.28	2 (0,6)	2 (0,4)	4 (0,10)
Perceived social support	1	—	—	—	—
Family decision-making	0.100*⁣*^*∗∗*^	1	—	—	—
Depression	−0.284*⁣*^*∗∗∗*^	−0.129*⁣*^*∗∗*^	1	—	—
Anxiety	−0.229*⁣*^*∗∗∗*^	−0.129*⁣*^*∗∗*^	0.596*⁣*^*∗∗∗*^	1	—
Stress	−0.284*⁣*^*∗∗∗*^	−0.122*⁣*^*∗∗*^	0.619*⁣*^*∗∗∗*^	0.574*⁣*^*∗∗∗*^	1

*Note:* Perceived social support and family decision-making using baseline data and depression, anxiety, and stress using follow-up data; depression, anxiety, and stress scores were positively skewed and therefore expressed using the interquartile range.

⁣^*∗∗*^*p* < 0.05.

⁣^*∗∗∗*^*p* < 0.001.

**Table 3:** Results of longitudinal causal mediation analysis(*N* = 331).

Effect type	Component	Depression			Anxiety			Stress		
		*β*	95% CI		*β*	95% CI		*β*	95% CI	
TE		**−1.323**	**−2.234**	**−0.412**	**−0.928**	**−1.603**	**−0.253**	**−1.351**	**−2.397**	**−0.305**
PNDE	CDE	**−1.035**	**−1.918**	**−0.152**	**−0.752**	**−1.418**	**−0.086**	**−1.029**	**−2.041**	**−0.017**
	RIE	−0.006	−0.076	0.065	−0.026	−0.095	0.044	−0.032	−0.126	0.063
TNIE	MIE	0.013	−0.150	0.177	0.059	−0.084	0.202	0.075	−0.120	0.269
	PIE	**−0.295**	**−0.587**	**−0.004**	**−0.210**	**−0.411**	**−0.008**	**−0.365**	**−0.725**	**−0.005**
P_PIE		22.33%			22.60%			27.02%		
OP_M		21.31%			16.23%			21.47%		

*Note*: The models controlled for the effects of confounding factors such as infant sex, number of infant siblings, infant delivery, maternal age, marital status, occupation, education, mother's work experience outside the home, whether the mother's natal family is in this township, whether the father lives together with the mother, number of adults at home, and family assets. The bold values indicate statistically significant results at the 0.05 level.

Abbreviations: OP_M, overall proportion mediated (TNIE/TE); P_PIE, proportion pure indirect effect (PIE/TE).

## Data Availability

The data that support the findings of this study are available from the corresponding author upon reasonable request.

## Nomenclature

CDE: Conditional direct effect
DAG: Directed acyclic graphs
DASS-21: The depression anxiety stress scale-21
IQR: Interquartile range
LMIC: Low-and middle-income countries
MIE: Mediated interaction effect
OP_M: Overall proportion mediated (TNIE/TE)
PIE: Pure indirect effect
P_PIE: Proportion pure indirect effect (PIE/TE)
RIE: Reference interaction effect
SD: Standard deviation
TE: Total effect
PNDE: Pure natural direct effect
TNIE: Total natural indirect effect
WHO: World Health Organization.

## Supporting Information

Supporting Information 1 Table S1–S5. Table S1: Family Decision-Making Scale Items. Table S2: Total Variance Explained by Extracted Components in the EFA. Table S3: Rotated Component Matrix from Principal Component Analysis. Table S4: Confirmatory Factor Analysis Results for the Family Decision-Making Scale. Table S5: Internal Consistency of the Family Decision-Making Scale.

Supporting Information 2 Table S6–S10. Table S6: Results of longitudinal causal mediation analysis in younger group (≤11 months, *N* = 142). Table S7: Results of longitudinal causal mediation analysis in older group (≥11 months, *N* = 189). Table S8: Causal mediation sensitivity analyses (*N* = 331). Table S9: E-values for longitudinal causal mediation effects. Table S10: Robustness check of longitudinal causal mediation analysis with binary family decision-making index (*N* = 331).
