# Supplementary material for: Role of Family Decision-Making and Perceived Social Support in the Mental Health of Mothers of Infants in Rural Western China
Source: Depress Anxiety. 2025 Nov 14;2025:8794211. doi: 10.1155/da/8794211 (PMC12638170; doi:10.1155/da/8794211)
Supplement: Supporting Information 1 — Table S1–S5. Table S1: Family Decision-Making Scale Items. Table S2: Total Variance Explained by Extracted Components in the EFA. Table S3: Rotated Component Matrix from Principal Component Analysis. Table S4: Confirmatory Factor Analysis Results for the Family Decision-Making Scale. Table S5: Internal Consistency of the Family Decision-Making Scale. [file 8794211.f1.docx]

**Appendix**

**Table S1: Family Decision-Making Scale Items**

Who in your family usually has the final say in decisions related to…

| Subscale | Item Content | Respondent | Jointly with others in household | Others in the household |
| --- | --- | --- | --- | --- |
| Decisions about family matters | 1.What food to buy for family meals? | 2 | 1 | 0 |
|  | 2. Whether to purchase small household items, such as utensils, lamps, etc.? | 2 | 1 | 0 |
|  | 3. Whether to purchase major goods for the household such as TVs, furniture, etc.? | 2 | 1 | 0 |
|  | 4. How the household earnings are spent? | 2 | 1 | 0 |
| Decisions about the mother’s personal matters | 5.Whether or not you should work to earn money? | 2 | 1 | 0 |
|  | 6. Obtaining healthcare for yourself? | 2 | 1 | 0 |
| Decisions about caring for young children | 7. Whether to exclusively breastfeed newborn for 6 months? | 2 | 1 | 0 |
|  | 8. What foods to feed the child? | 2 | 1 | 0 |
|  | 9. What to do if the child falls sick? | 2 | 1 | 0 |
|  | 10. How much to spent on healthcare for child? | 2 | 1 | 0 |

**Exploratory and Confirmatory Factor Analyses of the Family Decision-Making Scale**

**Exploratory factor analysis**

An exploratory factor analysis using principal component analysis with varimax rotation extracted three components with eigenvalues greater than 1, consistent with the theoretical structure. The KMO value was 0.787 and Bartlett’s test was significant (*p* < 0.001), indicating sampling adequacy. The three components explained 54.5% of the total variance after rotation. See Table S2 for details.

**Table S2. Total Variance Explained by Extracted Components in the EFA**

| Component | Initial Eigenvalues | | | Extraction Sums of Squared Loadings | | | Rotation Sums of Squared Loadings | | |
| --- | --- | --- | --- | --- | --- | --- | --- | --- | --- |
|  | Total | % of Variance | Cumulative % | Total | % of Variance | Cumulative % | Total | % of Variance | Cumulative % |
| 1 | 2.929 | 29.291 | 29.291 | 2.929 | 29.291 | 29.291 | 2.224 | 22.242 | 22.242 |
| 2 | 1.507 | 15.072 | 44.363 | 1.507 | 15.072 | 44.363 | 1.937 | 19.366 | 41.609 |
| 3 | 1.016 | 10.163 | 54.526 | 1.016 | 10.163 | 54.526 | 1.292 | 12.917 | 54.526 |
| 4 | 0.875 | 8.747 | 63.273 |  |  |  |  |  |  |
| 5 | 0.730 | 7.298 | 70.571 |  |  |  |  |  |  |
| 6 | 0.678 | 6.781 | 77.353 |  |  |  |  |  |  |
| 7 | 0.642 | 6.422 | 83.774 |  |  |  |  |  |  |
| 8 | 0.578 | 5.780 | 89.555 |  |  |  |  |  |  |
| 9 | 0.540 | 5.401 | 94.956 |  |  |  |  |  |  |
| 10 | 0.504 | 5.044 | 100.000 |  |  |  |  |  |  |

A principal component analysis was conducted using varimax rotation with Kaiser normalization to explore the underlying structure of the 10 items assessing family decision-making. The rotated component matrix revealed a three-factor solution that was consistent with the pre-specified conceptual domains: (1) decisions about family matters, (2) decisions about the mother’s personal matters, and (3) decisions about caring for young children. Each item loaded strongly on its corresponding component. The factor loadings are presented in Table S3.

**Table S3. Rotated Component Matrix from Principal Component Analysis**

|  | Component | | |
| --- | --- | --- | --- |
| Item No. | 1 | 2 | 3 |
| Item 1 |  | 0.642 |  |
| Item 2 |  | 0.764 |  |
| Item 3 |  | 0.667 |  |
| Item 4 |  | 0.644 |  |
| Item 5 |  |  | 0.792 |
| Item 6 |  |  | 0.673 |
| Item 7 | 0.704 |  |  |
| Item 8 | 0.737 |  |  |
| Item 9 | 0.732 |  |  |
| Item 10 | 0.695 |  |  |

**Confirmatory factor analysis**

A confirmatory factor analysis (CFA) was conducted to assess the construct validity of the 10-item family decision-making scale. The model demonstrated a good fit to the data: χ²/df = 2.72, GFI = 0.977, AGFI = 0.960, CFI = 0.955, TLI = 0.934, RMSEA = 0.048, and SRMR = 0.038. Standardized factor loadings ranged from 0.39 to 0.66, with most items loading above 0.60 (see Table S4 for details).

The average variance extracted (AVE) was 0.34, and the composite reliability (CR) was 0.84. While the AVE was below the commonly recommended threshold of 0.50, a CR above 0.70 is generally considered sufficient to indicate construct reliability, and the scale can still be regarded as having adequate psychometric quality [1]. The Cronbach’s α coefficient for the overall scale was 0.689, and item-deleted α values ranged from 0.644 to 0.697, supporting the internal reliability of the measure. Detailed factor loadings and reliability coefficients are presented in Table S5.

**Table S4. Confirmatory Factor Analysis Results for the Family Decision-Making Scale**

| Item No. | Standardized Factor Loading | AVE | CR |
| --- | --- | --- | --- |
| Item 1 | 0.41 | 0.34 | 0.84 |
| Item 2 | 0.59 |  |  |
| Item 3 | 0.60 |  |  |
| Item 4 | 0.61 |  |  |
| Item 5 | 0.39 |  |  |
| Item 6 | 0.66 |  |  |
| Item 7 | 0.60 |  |  |
| Item 8 | 0.64 |  |  |
| Item 9 | 0.63 |  |  |
| Item 10 | 0.63 |  |  |

**Table S5. Internal Consistency of the Family Decision-Making Scale**

| Item No. | Cronbach’s Alpha if Item Deleted | Total Cronbach’s Alpha |
| --- | --- | --- |
| Item 1 | 0.683 |  |
| Item 2 | 0.672 |  |
| Item 3 | 0.660 |  |
| Item 4 | 0.661 |  |
| Item 5 | 0.697 | 0.689 |
| Item 6 | 0.675 |  |
| Item 7 | 0.670 |  |
| Item 8 | 0.650 |  |
| Item 9 | 0.648 |  |
| Item 10 | 0.644 |  |

Taken together, the exploratory and confirmatory factor analyses support the structural validity of the scale, and the reliability indices indicate acceptable internal consistency. The scale can be reliably used to assess decision-making patterns among mothers of infants in rural western China.

**References**

[1]. Fornell C, Larcker DF. Evaluating structural equation models with unobservable variables and measurement error. J Mark Res. 1981 Feb;18(1):39–50. doi:10.2307/3151312.
