# Supplementary material for: Role of Family Decision-Making and Perceived Social Support in the Mental Health of Mothers of Infants in Rural Western China
Source: Depress Anxiety. 2025 Nov 14;2025:8794211. doi: 10.1155/da/8794211 (PMC12638170; doi:10.1155/da/8794211)
Supplement: Supporting Information 2 — Table S6–S10. Table S6: Results of longitudinal causal mediation analysis in younger group (≤11 months, N = 142). Table S7: Results of longitudinal causal mediation analysis in older group (≥11 months, N = 189). Table S8: Causal mediation sensitivity analyses (N = 331). Table S9: E-values for longitudinal causal mediation effects. Table S10: Robustness check of longitudinal causal mediation analysis with binary family decision-making index (N = 331). [file 8794211.f2.docx]

**Table S6. Results of longitudinal causal mediation analysis in younger group (≤11 months, N=142).**

|  | | Depression | | |  | Anxiety | | |  | Stress | | |
| --- | --- | --- | --- | --- | --- | --- | --- | --- | --- | --- | --- | --- |
|  |  | *β* | 95%*CI* | |  | *β* | 95%*CI* | |  | *β* | 95%*CI* | |
| TE | | **-1.999** | **-3.305** | **-0.693** |  | **-1.294** | **-2.326** | **-0.261** |  | **-1.983** | **-3.482** | **-0.485** |
| PNDE | CDE | **-1.643** | **-2.876** | **-0.411** |  | **-1.172** | **-2.183** | **-0.162** |  | **-1.625** | **-3.054** | **-0.196** |
|  | RIE | 0.001 | -0.027 | 0.027 |  | 0.001 | -0.076 | 0.078 |  | -0.001 | -0.074 | 0.072 |
| TNIE | MIE | -0.026 | -0.24 | 0.188 |  | 0.07 | -0.119 | 0.259 |  | -0.065 | -0.307 | 0.178 |
|  | PIE | -0.33 | -0.795 | 0.134 |  | -0.192 | -0.491 | 0.107 |  | -0.292 | -0.748 | 0.163 |

**Table S7. Results of longitudinal causal mediation analysis in older group (≥12 months, N=189).**

|  | | Depression | | |  | Anxiety | | |  | Stress | | | |
| --- | --- | --- | --- | --- | --- | --- | --- | --- | --- | --- | --- | --- | --- |
|  |  | *β* | 95%*CI* | |  | *β* | 95%*CI* | |  | *β* | 95%*CI* | |  |
| TE | | -0.894 | -2.154 | 0.366 |  | -0.509 | -1.404 | 0.386 |  | -0.931 | -2.373 | 0.51 |  |
| PNDE | CDE | -0.613 | -1.844 | 0.618 |  | -0.311 | -1.194 | 0.572 |  | -0.545 | -1.94 | 0.849 |  |
|  | RIE | -0.031 | -0.211 | 0.148 |  | -0.03 | -0.173 | 0.113 |  | -0.16 | -0.444 | 0.124 |  |
| TNIE | MIE | 0.045 | -0.211 | 0.302 |  | 0.045 | -0.167 | 0.257 |  | 0.23 | -0.151 | 0.611 |  |
|  | PIE | -0.295 | -0.685 | 0.095 |  | -0.213 | -0.483 | 0.057 |  | -0.457 | -1.006 | 0.092 |  |

**Table S8. Causal mediation sensitivity analyses (N=331).**

|  | | Depression | | |  | Anxiety | | |  | Stress | | |
| --- | --- | --- | --- | --- | --- | --- | --- | --- | --- | --- | --- | --- |
|  |  | *β* | 95%*CI* | |  | *β* | 95%*CI* | |  | *β* | 95%*CI* | |
| TE | | **-1.339** | **-2.25** | **-0.428** |  | **-0.911** | **-1.586** | **-0.236** |  | **-1.371** | **-2.41** | **-0.331** |
| PNDE | CDE | **-1.049** | **-1.933** | **-0.165** |  | **-0.731** | **-1.396** | **-0.065** |  | **-1.05** | **-2.057** | **-0.043** |
|  | RIE | -0.006 | -0.077 | 0.065 |  | -0.026 | -0.096 | 0.044 |  | -0.034 | -0.13 | 0.063 |
| TNIE | MIE | 0.013 | -0.152 | 0.179 |  | 0.06 | -0.084 | 0.205 |  | 0.079 | -0.118 | 0.276 |
|  | PIE | **-0.297** | **-0.589** | **-0.006** |  | **-0.215** | **-0.419** | **-0.011** |  | **-0.366** | **-0.722** | **-0.010** |
| P_PIE | | 22.22% | | |  | 23.57% | | |  | 26.70% | | |
| OP_M | | 21.21% | | |  | 16.95% | | |  | 20.96% | | |

Note: Infant's age, infant's sex, number of infant siblings, infant delivery, mother's age, marital status, occupation, education, mothers' work experience outside, whether the mother's natal family is in this township, whether the father lives together, number of adults at home, and family assets as covariates in the adjusted model.

**Table S9. E-values for longitudinal causal mediation effects.**

| Outcome | Effect | *β* | 95% CI | | OR | E-value | E-value (CI- limit) |
| --- | --- | --- | --- | --- | --- | --- | --- |
| Depression | TE | -1.323 | -2.234 | -0.412 | 0.266 | 6.97 | 2.39 |
|  | CDE | -1.035 | -1.918 | -0.152 | 0.355 | 5.08 | 1.60 |
|  | PIE | -0.295 | -0.587 | -0.004 | 0.745 | 2.02 | 1.07 |
| Anxiety | TE | -0.928 | -1.603 | -0.253 | 0.395 | 4.50 | 1.90 |
|  | CDE | -0.752 | -1.418 | -0.086 | 0.471 | 3.66 | 1.40 |
|  | PIE | -0.21 | -0.411 | -0.008 | 0.811 | 1.77 | 1.10 |
| Stress | TE | -1.351 | -2.397 | -0.305 | 0.259 | 7.19 | 2.05 |
|  | CDE | -1.029 | -2.041 | -0.017 | 0.357 | 5.04 | 1.15 |
|  | PIE | -0.365 | -0.725 | -0.005 | 0.694 | 2.24 | 1.08 |

Note: a. ORs were obtained as OR=exp(β).

b. E-values were computed on the risk-ratio scale as $E=R+\sqrt{R\times(R-1)}$, where 𝑅 is the relative effect (RR/HR, or OR when appropriate). For protective effects (𝑅<1), we used $1/R$.

c. CI-limit E-value was calculated using the 95% CI bound closest to 1 with the same formula; it quantifies the minimum confounder–exposure and confounder–outcome associations (risk-ratio scale) required to move the 95% CI to include 1 and thus serves as a conservative robustness metric.

d. RIE/MIE were not displayed because their contributions to the total effect were minimal (≤6.4%) and statistically non-significant.

**Table S10.** **Robustness check of longitudinal causal mediation analysis with binary family decision-making index (N=331)**

|  | | Depression | | |  | Anxiety | | |  | Stress | | |
| --- | --- | --- | --- | --- | --- | --- | --- | --- | --- | --- | --- | --- |
|  |  | *β* | 95%*CI* | |  | *β* | 95%*CI* | |  | *β* | 95%*CI* | |
| TE | | **-1.419** | **-2.154** | **-0.683** |  | **-0.696** | **-1.243** | **-0.149** |  | **-1.157** | **-1.997** | **-0.317** |
| PNDE | CDE | **-1.156** | **-1.860** | **-0.451** |  | **-0.537** | **-1.073** | **-0.002** |  | **-0.882** | **-1.692** | **-0.071** |
|  | RIE | 0.023 | -0.044 | 0.090 |  | 0.012 | -0.027 | 0.051 |  | 0.009 | -0.036 | 0.055 |
| TNIE | MIE | -0.080 | -0.214 | 0.055 |  | -0.039 | -0.139 | 0.060 |  | -0.033 | -0.167 | 0.102 |
|  | PIE | **-0.207** | **-0.405** | **-0.008** |  | **-0.131** | **-0.261** | **-0.001** |  | **-0.252** | **-0.493** | **-0.010** |
| P_PIE | | 14.56% | | |  | 18.78% | | |  | 21.76% | | |
| OP_M | | 20.17% | | |  | 24.44% | | |  | 24.60% | | |

Note：The models also controlled for the effects of confounding factors such as infant sex, number of infant siblings, infant delivery, maternal age, marital status, occupation, education, mother’s work experience outside the home, whether the mother's natal family is in this township, whether the father lives together with the mother, number of adults at home, and family assets. P_PIE: proportion pure indirect effect (PIE/TE); OP_M: overall proportion mediated (TNIE/TE).
